# Gallium extraction from Bayer liquor using hydroxamic acid-functionalized poly(ethyl acrylate) adsorbents with oriented porous structures

**DOI:** 10.1039/d6ra05324f

**Published:** 2026-08-19

**Authors:** Li Ren, Wei Liu, Xian Wu, Yanbin Tu, Zhengjie Yao, Yang Li

**Affiliations:** a School of Materials and Energy Engineering, Guizhou Institute of Technology Guiyang China 20130214@git.edu.cn

## Abstract

As a waste resource from the aluminum industry, Bayer liquor contains a large amount of gallium (Ga) ions. The recovery of Ga from Bayer liquor is of great significance for resource utilization and obtaining high-value metals. Poly(ethyl acrylate) (PEA) with an oriented porous structure was prepared by the ice-templating method. Hydroxamic acid groups were grafted onto the pore surfaces to construct specific adsorption sites, forming efficient mass transfer channels and abundant specific functional groups. The novel PEA adsorbent, which contained regular vertically arranged oriented pores, had a specific surface area as large as 10.96 m^2^ g^−1^ available for metal adsorption. Its rich hydroxamic acid groups were evenly distributed within the adsorbent. The extensive chelation of Ga(iii) was achieved on the surface of the pores, exhibiting excellent adsorption performance. In the simulated Bayer liquor, the equilibrium adsorption capacity for Ga(iii) reached 27.35 mg g^−1^. The adsorption followed a chemisorption mechanism with homogeneous monolayer characteristics. The porous PEA adsorbents also exhibited excellent adsorption performance over a wide concentration range. In the real Bayer liquor containing coexisting ions such as vanadium, the equilibrium capacity slightly decreased to 19.46 mg g^−1^ due to competitive adsorption. This work provides a novel strategy for designing high-performance porous adsorbents for Ga recovery in complex industrial wastewater systems.

## Abstract 2

As a waste resource from the aluminum industry, Bayer liquor contains a large amount of gallium (Ga) ions. The recovery of Ga from Bayer liquor is of great significance for resource utilization and obtaining high-value metals.

## Graphical abstract

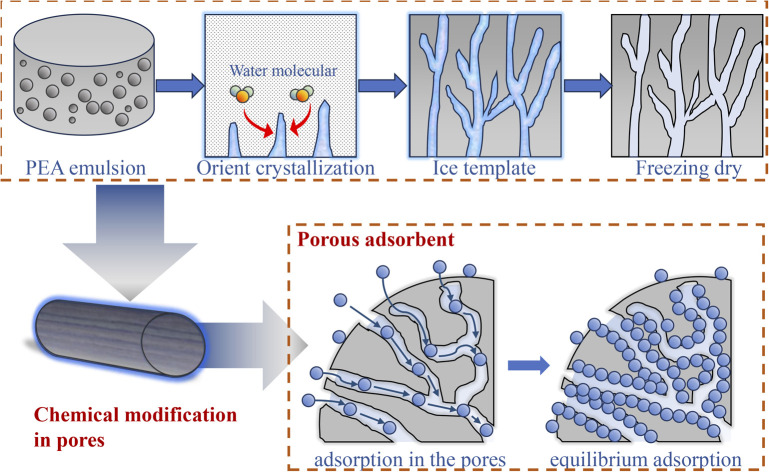

## 1 Introduction

Gallium (Ga) metal is an important strategic resource that is crucial for manufacturing semiconductor, optoelectronic, and renewable energy devices.^1^ Ga metal exhibits distinctive physical properties, with a melting point of 29.78 °C and a boiling point of 2403 °C. It has the widest liquid-phase temperature range among all natural metals.^2^ Since then, it has been an ideal material for high-temperature measurement and heat transfer. Its compounds, such as GaAs,^3^ GaN,^4^ and Ga_2_O_3_,^5^ can be used as core components for solar cells, photodetectors, and space electronics with superior radiation resistance due to their high electron mobility, wide bandgap and excellent optoelectronic performance. However, Ga is a typically rare and dispersed metal. Over 99% of the world's Ga is present in the form of isomorphic minerals in bauxite and lead–zinc deposits, which cannot form independent and exploitable mineral deposits.^6^ Currently, the world's primary production of Ga comes from the by-product of the Bayer process for producing alumina, *i.e.* Bayer liquor, with the remaining small amount coming from zinc smelting waste residue.^7^ The efficient extraction of Ga from the high-salinity and complex cation coexisting systems of industrial waste is of great significance for resource sustainability and economic feasibility, but it still remains a major challenge.^8^

Consequently, its extraction pathway is highly dependent on the aluminum industry.^9^ The Ga(iii) content varies significantly in different bauxites in various places around the world. When the Ga(iii) content in bauxite is low, the extraction cost is significantly increased. As a major aluminum-producing area in the world, China has a large amount of Ga resources in its bauxites. With the rapid development of the electronics industry and international resource management policies, the price of Ga resources has been constantly increasing. However, the Ga content in the Bayer liquor is considered an unrecoverable impurity due to its low concentration of only 0.1–0.3 g L^−1^. The chemical properties of Ga are similar to those of aluminum, and it is often discharged and discarded along with red mud. With technological advancements, many useful recycling processes have been developed, and the waste liquor has received renewed attention as an important source of Ga resources.^10^

The existing adsorbents for Ga extraction include ion exchange resins,^11^ porous carbon,^12^ graphene,^13^ COF,^14^ mixed matrix membranes,^15^ and some inorganic materials.^16^ Many of them have limited adsorption capacity, slow adsorption rate, and high production cost. The most critical issue was the poor selectivity in the presence of a coexisting cation system.^17^ Vanadium(v) exhibits a similar chemical affinity to Ga(iii) and thus competes fiercely for adsorption active sites, severely reducing the selectivity of conventional adsorbents. In addition, the mass transfer and diffusion limitations caused by traditional adsorptive structures resulted in unsatisfactory Ga recovery efficiency in actual industrial systems.^18^

Recently, research has focused on developing novel adsorbents with a combination of optimized structures and specific functional groups to overcome the aforementioned limitations.^19,20^ In terms of structural design, constructing oriented pores can significantly shorten the diffusion path and improve the accessibility of internal adsorption sites, thus enhancing the mass transfer efficiency and adsorption kinetics.^21^ In terms of chemical modification, hydroxamic acid derivatives are efficient chelating groups that enhance the binding ability and selectivity towards target metal ions.^22^ Hydroxamic acid can be regarded as a derivative of carboxylic acid and exhibits strong chelating ability for metal ions.^23^ Due to its unique structure, hydroxamic acid groups can chelate molecules to form a five-membered ring complex. Guo *et al.* prepared an adsorbent with a size of approximately 50 µm by modifying chitin with hydroxamic acid, achieving a maximum adsorption capacity of 118.22 mg g^−1^, and the adsorption efficiency remained at 80% even after 5 cycles.^24^ Cao *et al.* prepared a porous poly (6-acrylylamino-hexyl hydroxamic acid) resin with a wrinkled surface, which achieved adsorption capacities of 1.030, 0.962, and 1.450 mmol g^−1^ for La(iii), Ce(iii), and Y(iii), respectively.^25^ Zhao *et al.* synthesized a polypropylene–hydroxylamine hydrogel through free radical polymerization at room temperature.^26^ The maximum adsorption capacity of the adsorbent for Ga(iii) reached 156.13 mg g^−1^, and it exhibited good competitive adsorption performance in Zn(ii), Cu(ii), Al(iii), and In(iii) environments. Besides, Li *et al.* used a polyacrylic acid resin as the matrix and introduced hydroxylamine groups to obtain an adsorbent with a nitrogen content of 3.85%.^27^ This adsorbent achieved a maximum adsorption capacity of 27.65 mg g^−1^ for Ga(iii). For an inorganic adsorbent, Lu *et al.* modified the surface of spherical SiO_2_ with polyacrylonitrile and divinylbenzene hydroxylamine groups, achieving a Ga(iii) adsorption capacity of 1.34 mmol g^−1^.^28^ Its desorption was completed within 45 min using a 1.5 M HCl solution. Liu *et al.* utilized micrometer sized CaCO_3_ as a hard template to manufacture a macroporous cellulose bead adsorbent,^29^ and hydroxamic acid groups were introduced into the porous adsorbent. This adsorbent removed various heavy metals from water. The maximum adsorption capacities of Cu(ii), Mn(ii), and Ni(ii) reached 346.5, 261.5, and 243.2 mg g^−1^, respectively.

Although hydroxamic acid-functionalized materials have proven effective for metal ion adsorption, most reported adsorbents suffer from either uncontrollable porous structures or low grafting density of functional groups. It remains challenging to simultaneously optimize the pore structure and surface chemical properties of a single adsorbent. Against this background, we propose a novel adsorbent design strategy combining oriented porous structures and high-density hydroxamic acid groups. The present study aimed to propose a novel design strategy for an adsorbent that simultaneously optimizes the pore structure and surface chemical properties. In this work, poly(ethyl acrylate) (PEA) was chosen as the raw material for the adsorbent. PEA was synthesized *via* emulsion polymerization. Each of its monomers contained an ethyl acrylate group.^30^ The positively charged carbonyl carbon atom in the ethyl acrylate group was attacked by the nitrogen atom in hydroxylamine, forming hydroxamic acid through a nucleophilic addition reaction. Consequently, it endowed PEA with a large number of functional groups. Furthermore, since PEA was synthesized by emulsion polymerization, its oil/water environment formed an ideal aqueous polymer solution. By employing the ice-templating oriented crystallization method combined with freeze-drying to remove ice, adsorbents with oriented pores could be obtained. This unique structure provided short diffusion paths and a high specific surface area, while the introduced hydroxamic acid groups served as selective chelating sites for Ga(iii), which enhanced both the number and availability of chelating sites between Ga(iii) and hydroxamic acid groups. Experiments were conducted by performing isothermal adsorption in simulated solutions and competitive adsorption of V in the real Bayer liquor. This work mainly focused on the application potential of a structure and function-integrated adsorbent in Ga recovery, which is expected to provide a new pathway for the efficient and highly selective recovery of metals from complex industrial waste.

## 2 Materials and methods

### 2.1 Materials

The chemicals used for PEA preparation, ethyl acrylate (EA), polyvinyl alcohol (PVA), sodium dodecylbenzene sulfonate (SDS), and ammonium persulphate (APS), were purchased from Shanghai Aladdin Biochemical Technology, China. The reagents used for hydroxamic acid modification, hydroxylamine hydrochloride and sodium hydroxide, were obtained from Shanghai Aladdin Biochemical Technology, China. The simulated solution, gallium sulfate hydrate, and analytical reagent were also purchased from Shanghai Aladdin Biochemical Technology, China. The real Bayer liquor was obtained from an aluminum plant in Zunyi, Guizhou Province, China.

### 2.2 Preparations

#### 2.2.1 Synthesis of the PEA emulsion

**Fig. 1:**
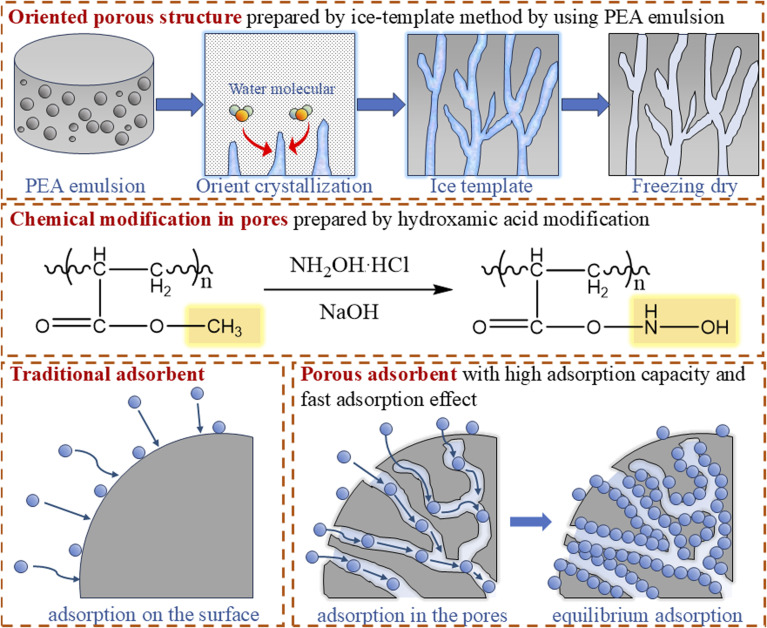
Schematic of the fabrication of the oriented porous PEA, hydroxamic acid modification, and comparison of the adsorption mechanism between conventional adsorbents and the as-prepared A-PEA.

The synthesis route is illustrated in Fig. 1. The polymerization was initiated by introducing 100 g of EA monomer and 500 mL deionized water. 1 g of PVA was dissolved in deionized water in a flask equipped with a mechanical stirrer and a reflux condenser. PVA served as the emulsifier and stabilizer in the emulsion polymerization of ethyl acrylate. It reduced interfacial tension, stabilized the monomer droplets, and prevented the coagulation of the polymer particles during polymerization. The solution was heated to 90 °C for 30 min. Then, the surfactant SDS with a concentration of 0.5 wt% was added to the solution. Subsequently, the initiator of APS with a concentration of 0.2 wt% was dissolved in deionized water and added to the reaction flask. The heating temperature of the flask was adjusted to 65 °C. After 2 h of emulsion pre-polymerization, the reaction solution was gradually heated to 90 °C and maintained for 2 h to complete polymerization. The solution was obtained and designated as the PEA emulsion.

#### 2.2.2 Preparation of the oriented porous PEA

The PEA emulsion was poured into a polytetrafluoroethylene (PTFE) mold, which was placed on a copper cold finger in a homemade liquid-nitrogen pool. The temperature of the cold finger was set to −120 °C to induce stable oriented freezing. As depicted in Fig. 1, ice nucleation was initiated at the surface of the cold finger and propagated upward, forming vertically oriented crystals that templated the pore structure. The isothermal crystallization time was adjusted to 30 min to ensure the complete crystallization of water in the PEA emulsion. Subsequently, the frozen PEA solutions were removed from the PTFE mold and placed in a glass Petri dish. Finally, the Petri dish containing the sample was placed inside a freeze dryer (LGJ18, Ningbo Xinyi, China) at −70 °C for 24 h to remove the ice, yielding the oriented porous PEA.

#### 2.2.3 Preparation of the hydroxamic acid-modified PEA adsorbent

Hydroxamic acid modification on the inner pore walls of PEA was performed following a literature-reported procedure.^31^ First, 10 g of porous PEA was added to 300 mL of butanol under stirring for 10 min. Subsequently, 4 g of NH_2_OH·HCl was added to the solution, which was then stirred at 75 °C in an oil bath for 24 h. Sodium hydroxide was gradually added to maintain the solution pH at 14 throughout the reaction. Sodium hydroxide reacted with hydroxylamine hydrochloride. The hydroxide ion derived from sodium hydroxide abstracted a proton from hydroxylamine hydrochloride, resulting in the formation of hydroxylamine, sodium chloride, and water. The next step was an aminolysis reaction, which is a type of nucleophilic substitution. The ester (–COOC_2_H_5_) group reacted with hydroxylamine, providing specific adsorption sites for the target molecules. After cooling to room temperature, the resulting material, named A-PEA, was washed with deionized water until the pH reached 8 and dried under vacuum at 70 °C for 4 h. An A-PEA adsorbent with hydroxamic acid groups was obtained. The lone pair of electrons on the nitrogen and oxygen atoms enabled the formation of stable chelates with a variety of metal ions, as shown in Fig. 1.

### 2.3 Characterizations

#### 2.3.1 Chemical composition analysis

Fourier transform infrared (FTIR; Nicolet 8700, Thermo Fisher Scientific, USA) spectroscopy and X-ray photoelectron spectroscopy (XPS; ESCALAB QXi, Thermo Fisher Scientific, USA) were employed to characterize the chemical elements and surface functional groups of the porous PEA adsorbents. FTIR spectra were collected in the range of 4000–400 cm^−1^ using the KBr pellet method, while XPS measurements were performed with an Al Kα X-ray source to study the chemical structure of the porous PEA adsorbents.

#### 2.3.2 Thermal analysis

The thermal stability of the porous PEA adsorbents was evaluated using a thermogravimetric analyzer (TGA; STA449F3, Netzsch, Germany). The samples were heated from room temperature to 700 °C at a heating rate of 20 °C min^−1^ under an N_2_ atmosphere. Differential scanning calorimetry (DSC; HP204F1, Netzsch, Germany) was used to analyze the thermal properties. The samples were heated from −30 °C to 130 °C at a rate of 10 °C min^−1^ and then cooled to −30 °C at the same rate. The tests were conducted under N_2_ atmosphere.

#### 2.3.3 Morphology and elemental distribution analysis

Scanning electron microscopy (SEM; Nova Nano 450, FEI, USA) was used to examine the surface morphology of the porous PEA adsorbents. The samples were sputter-coated with a thin layer of gold before imaging to improve their electrical conductivity. Energy-dispersive spectroscopy (EDS; EDAX, Ametek, USA) was coupled with the SEM to map the elemental composition and distribution on the surface of the adsorbents.

#### 2.3.4 Porous structure analysis

The N_2_ adsorption–desorption isotherms were measured at 77 K using a specific surface area and pore size analyzer (Autosorb iQ 2800TP, CIQTEK, China). Prior to testing, the samples were degassed at 80 °C for 12 h under vacuum to ? moisture. The Brunauer–Emmett–Teller (BET) method was applied to calculate the specific surface area, while the Barrett–Joyner–Halenda (BJH) model was used to determine the pore size distribution and total pore volume from the desorption isotherm.

#### 2.3.5 Adsorption experiments

To simulate industrial Bayer liquor, Ga(iii) solutions were prepared by dissolving Ga_2_(SO_4_)_3_·18H_2_O in deionized water. The solutions were adjusted to pH 14 by adding NaOH. Simulated Bayer liquors with different Ga(iii) concentrations of 40, 80, and 160 mg L^−1^ were prepared by dilution. The solid–liquid ratio was 1.0 g L^−1^. All adsorption experiments were performed in triplicate, and the average values were recorded.

Static adsorption experiments were conducted in 250 mL three-necked flasks. 100 mL of simulated Bayer liquor was transferred to each flask, followed by the addition of 0.2 g of the A-PEA adsorbent. The mixture was agitated in a constant-temperature water bath shaker at 25 °C for 20 h to ensure adsorption equilibrium was achieved. After adsorption, the adsorbent was separated from the solution using a 0.45 µm cellulose acetate membrane filter. The static cycling adsorption experiment was carried out under the same conditions. The adsorption medium was a simulated solution with a Ga concentration of 100 mg L^−1^ at pH 14 and eluted with 1.0 M HCl.

The Ga(iii) concentrations in the solutions before and after adsorption were determined using inductively coupled plasma optical emission spectrometry (ICP-OES; Avio 500, PerkinElmer, USA). The adsorption rate was calculated using the following equation:1
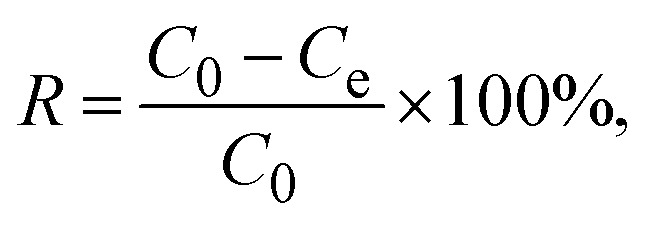
where *C*_0_ (mg L^−1^) is the initial Ga(iii) concentration, and *C*_e_ (mg L^−1^) is the equilibrium Ga(iii) concentration after adsorption.

The adsorption kinetics of the A-PEA adsorbent were studied using the pseudo-first-order kinetic and pseudo-second-order kinetic models. The pseudo-first-order kinetic model was expressed by eqn (2):

2 *q*_*t*_ = *q*_e_(1 − e^−*k*_1_*t*^),

where *q*_e_ and *q*_*t*_ are the adsorption capacities of Ga (mg g^−1^) at equilibrium and time *t* (min), respectively, and *k*_1_ is the rate constant of pseudo-first-order adsorption (min^−1^).

The pseudo-second order of the kinetic rate equation is expressed as eqn (3):3
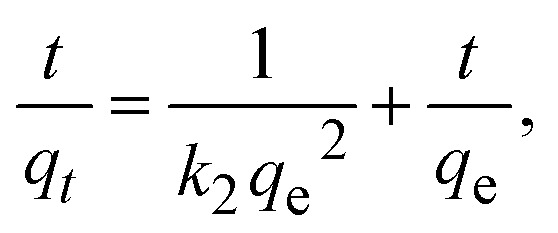
where *k*_2_ is the rate constant of pseudo-second-order adsorption (mg g^−1^ min^−1^).

The Langmuir and Freundlich models were commonly used to fit the adsorption isotherm models. Langmuir and Freundlich's equations are shown in eqn (4) and (5):4
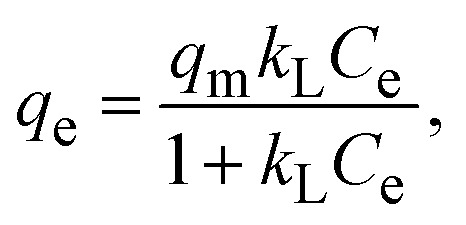
5
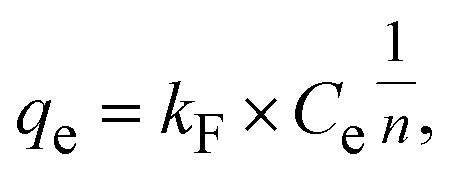
where *q*_m_ is the monolayer adsorbent capacity under equilibrium conditions (mg g^−1^), which indicates the maximum concentration retained by the adsorbent surface, and *K*_L_ is the Langmuir constant (L mg^−1^). *K*_F_ is the Freundlich constant related to the adsorption capacity, and 1/*n* is the intensity factor.

A static adsorption experiment in real Bayer liquor was also performed. The composition of the real Bayer liquor and the Ga(iii) concentration during adsorption were determined using ICP-OES.

## 3 Results and discussion

### 3.1 Chemical structure of the A-PEA adsorbent

**Fig. 2:**
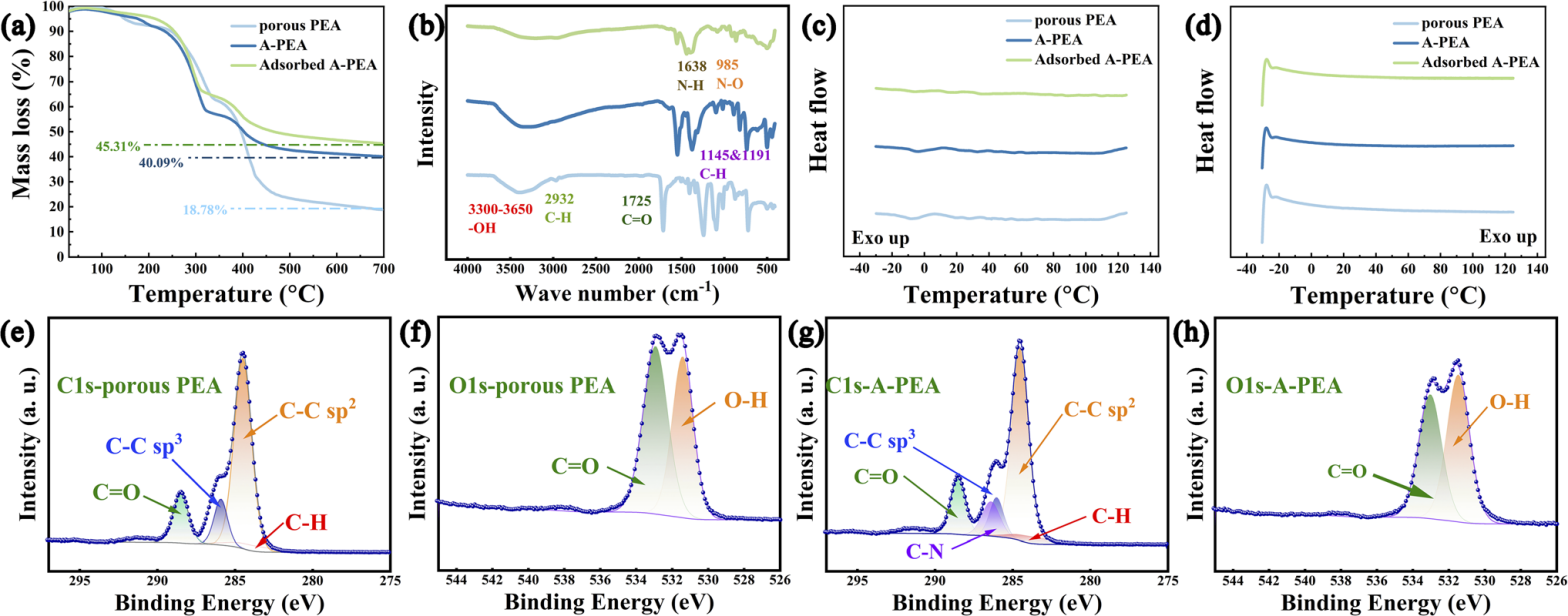
(a) TGA curves, (b) FTIR spectra, and DSC curves with a (c) cooling rate of 10 °C min^−1^ and (d) heating rate of 10 °C min^−1^ of porous PEA, A-PEA, and A-PEA after adsorption, (e) C 1s XPS spectra and (f) O 1s XPS spectra of porous PEA, and (g) C 1s XPS spectra and (h) O 1s XPS spectra of A-PEA.

Fig. 2 presents the characterizations of TGA, FTIR spectroscopy, DSC, and XPS for the oriented porous PEA prepared by the ice-templating method combined with hydroxamic acid group modification during its fabrication and adsorption processes. As shown in Fig. 2a, the TGA curve of the pristine PEA indicated an initial thermal decomposition temperature of 250 °C, with the mass loss exhibiting a single-stage characteristic corresponding to the thermal degradation of PEA chains, yielding a final residual carbon content of 18.78%. For A-PEA, the initial thermal decomposition temperature remained almost unchanged. However, an additional mass loss step emerged in the temperature range of 300 to 400 °C, which could be attributed to the thermal decomposition of the hydroxamic acid groups. In order to achieve high-efficiency adsorption, the adsorption temperature was adjusted to 30 °C.^32^ This observation also confirmed that the A-PEA adsorbent was thermally stable up to 70 °C, which was higher than the adsorption temperature. Concurrently, as shown in Fig. 2b, the FTIR spectrum of A-PEA showed the appearance of an N–H bending vibration peak at 1638 cm^−1^ and an N–O stretching vibration peak at 985 cm^−1^, providing clear evidence for the successful grafting of hydroxamic acid groups onto the surface of the pores. After chelation of Ga(iii), the TGA curve of A-PEA showed that the initial decomposition temperature increased to 270 °C, and the mass loss markedly increased to 45.58%, which was 16.80% higher than that of PEA. This enhancement in the final residual mass was attributed to the adsorption of cations and the coordination interaction between Ga(iii) and the hydroxamic acid groups. Furthermore, the FTIR spectra revealed a shift in the N–H vibration peak to 1630 cm^−1^, offering additional confirmation of the formation of Ga–N coordination bonds. As depicted in Fig. 2c and d, the DSC curve of pristine PEA exhibited a glass transition temperature (*T*_g_) of −15 °C, which was higher than the values reported in the literature.^33^ This could be attributed to the differences in molecular weight and crosslinking degree, which restricted chain mobility and elevated the glass transition temperature.^34^ After hydroxamic acid modification, the *T*_g_ value increased to −10 °C. This elevation was primarily due to the effects induced by the introduced hydroxamic acid groups, leading to enhanced intermolecular interactions and a reduction in chain segment mobility. Consequently, the chain segments required higher energy to undergo conformational changes, leading to an increase in *T*_g_. It should be noted that this change in thermal properties mainly induced a brittle-to-ductile transition of the adsorbent and had a slight impact on the adsorption process occurring above this temperature range. Moreover, the adsorbent maintained flexibility, preventing brittle fracture during solution flow.

As shown in Fig. 2e–h, the XPS analysis of the C 1s and O 1s spectra indicated that the surface of pristine PEA was composed of C, O, and H elements, featuring bonds such as C–C, C=O, and C–H. However, in the C 1s spectrum of A-PEA, a peak corresponding to C–N at 285.5 eV appeared, and the intensity of the C–O peak at 533.0 eV in the O 1s spectrum decreased. These spectral changes confirmed that the ester groups in PEA were consumed during the reaction, with the hydroxamic acid groups being grafted. Consequently, these characterizations demonstrated that hydroxamic acid modification was successfully achieved, and to some extent, adsorption was accomplished through the coordination interaction between Ga(iii) and the hydroxamic acid groups.

### 3.2 Morphology of the A-PEA adsorbent

**Fig. 3:**
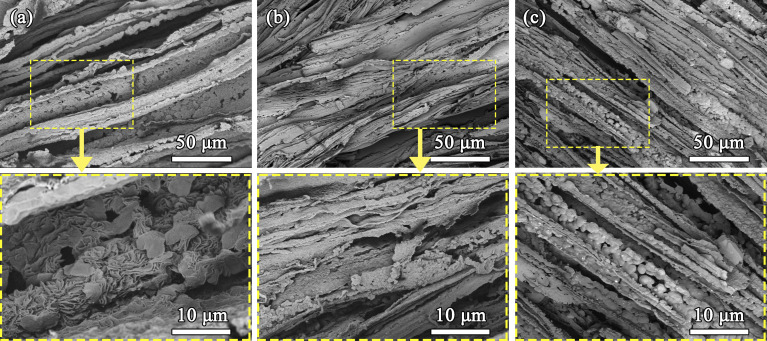
SEM images of (a) porous PEA, (b) A-PEA, and (c) A-PEA after adsorption.

Fig. 3 illustrates the morphological evolution of the PEA adsorbent during the chemical modification and adsorption processes, as characterized using SEM. The pristine porous PEA exhibited a well-defined, oriented, and straight channel with a characteristic pore size of ∼40 µm, as shown in Fig. 3a. Clearly, these channels were the residual traces left after the freeze-drying of the columnar ice formed by the oriented crystallization of the PEA emulsion.^35^ In detail, the oriented crystallization of water was induced through oriented freezing, forming columnar ice. During freeze-drying, the PEA resin was gradually separated from the emulsion and formed a framework with well-aligned channels, and this process controlled the structure and size of the pores. It is worth noting that the channel walls exhibited wrinkled features, which might be attributed to the shrinkage of PEA after ice sublimation; however, the main channel structure was still preserved. This micron-sized porous structure generated efficient channels for the rapid diffusion of cation-containing liquors due to the capillary effect,^36^ enabling rapid penetration without the need for gravity and external energy input.^37^ For A-PEA, as shown in Fig. 3b, after hydroxamic acid modification, the porous structure was still retained, and there was no significant change in its size. The structural stability was the result of the aminolysis reaction. Hydroxamic acid modification proceeded *via* the aminolysis of the ethyl ester groups on the PEA chains by hydroxylamine under alkaline conditions, where NaOH deprotonated hydroxylamine and facilitated the nucleophilic acyl substitution reaction. This process only acted on the pore surface without destroying the overall porous structure, thus maintaining the structural stability of the micron-sized pores. The most significant morphological change caused by this reaction was the densification and reconstruction of the pore wall surface. As shown in the high-magnification SEM image, the surface was observed to be smoother compared with that of pristine PEA, which might be due to local swelling caused by the alkaline reaction environment during hydroxamic acid modification and the dissolution of low-molecular-weight polymers.^38^ For A-PEA after adsorption, as shown in Fig. 3c, it was clear that the surface of the channels was filled with adsorbed particles with a size range from 1 µm to 3 µm. Although the pore size was slightly reduced due to the existence of adsorbed substances, the overall oriented structure was well-preserved. The synergistic effect of the oriented porous structure and surface functional modification of the PEA adsorbent not only shortened the diffusion path of the Ga-containing liquor but also provided stability in both the structure and chemical properties after desorption, offering a reusable and rapid pathway for designing metal-ion separation adsorbents.

**Fig. 4:**
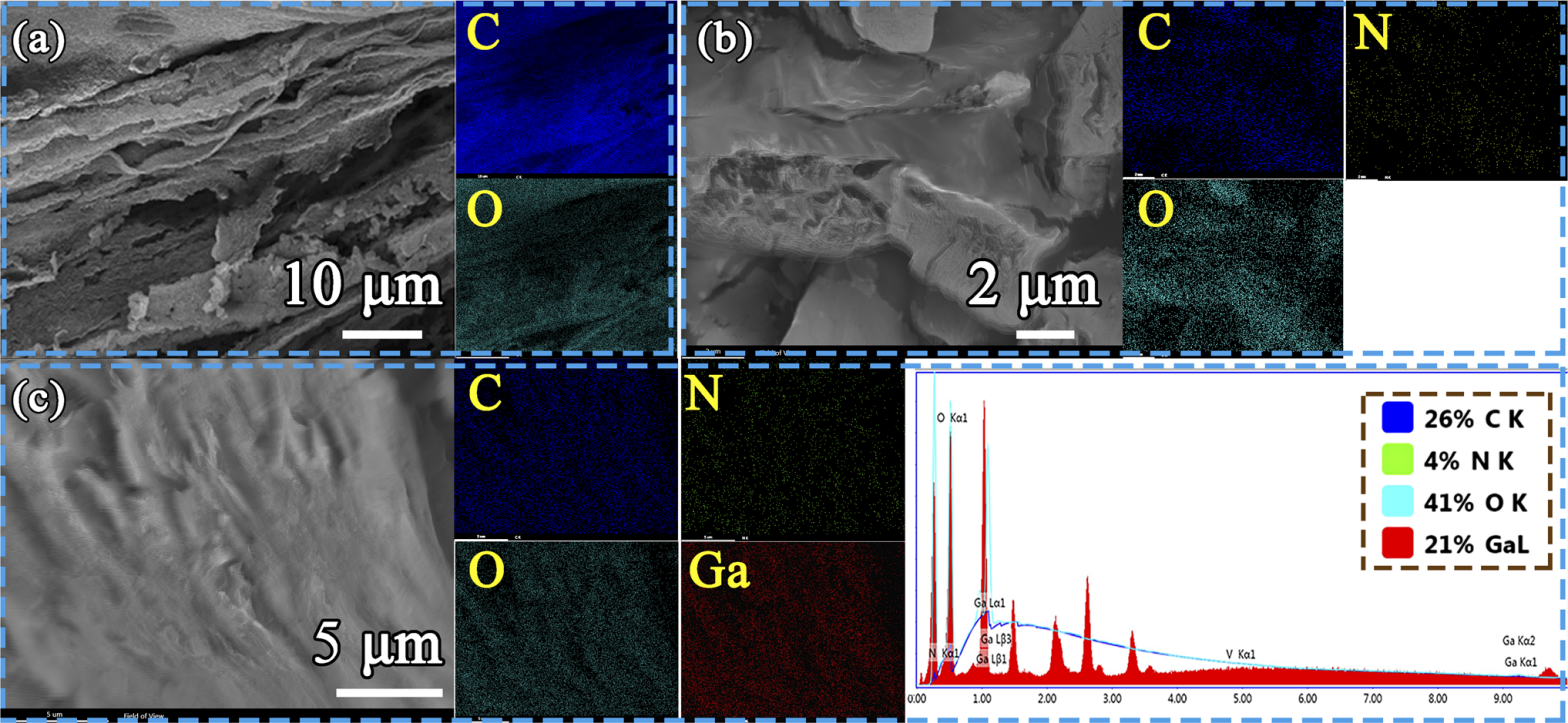
EDS elemental mapping images of (a) porous PEA, (b) A-PEA, and (c) A-PEA after adsorption.

To further prove that the particles in Fig. 3 contain Ga, Fig. 4 displays the evolution of element distribution for the PEA adsorbent characterized by EDS mapping, which enabled the visualization and analysis of the spatial distribution of different elements within the adsorbent. The EDS mapping of pristine PEA shown in Fig. 4a revealed the presence of only C and O elements, corresponding to its well-defined oriented porous structure. These two elements were uniformly distributed. This structure provided a foundation for subsequent functional modification for Ga recovery. For A-PEA in Fig. 4b, the N element was detected and distributed on the surface of the channels, while the distribution patterns of the C and O elements remained same. This not only confirmed the successful grafting of the hydroxamic acid groups onto the channel surfaces but also indicated that the oriented porous structure was intact during the modification process. In the case of A-PEA after adsorption, as shown in Fig. 4c, the Ga element was clearly observed, and Ga distribution showed an overlap with that of N elements. The quantitative analysis of the elements on the surface further revealed that the Ga content reached 21%, which confirmed that Ga was adsorbed onto the adsorbent's surface through the specific coordination interaction with the hydroxamic acid groups.

**Fig. 5:**
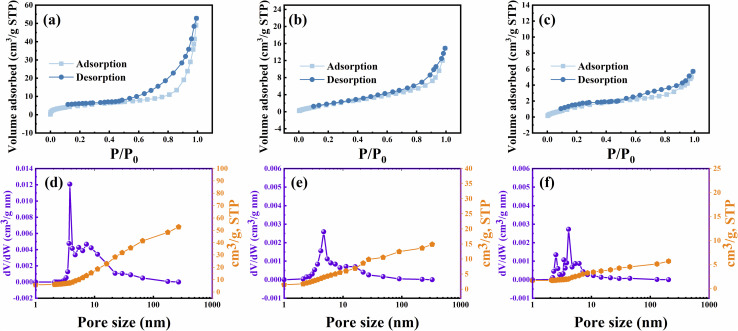
N_2_ adsorption–desorption curves of (a) porous PEA, (b) A-PEA, and (c) A-PEA after adsorption, and the pore size distribution curves of (d) porous PEA, (e) A-PEA, and (f) A-PEA after adsorption.

To evaluate the porous structural parameters of the PEA adsorbent, N_2_ adsorption/desorption tests were conducted. The BET specific surface area, pore size, and pore volume are presented in Fig. 5 and Table 1. As shown in Table 1, the oriented porous PEA possessed a BET specific surface area of 18.23 m^2^ g^−1^, an average pore size of 17.91 nm, and a total pore volume of 0.085 cm^3^ g^−1^. The structural parameters confirmed that the ice-templating method constructed an open porous structure with a high specific surface area and predominantly mesoporous characteristics. However, after hydroxamic acid modification, the BET specific surface area of the resulting A-PEA decreased to 10.96 m^2^ g^−1^, its average pore size was reduced to 11.19 nm, and its pore volume shrank to 0.058 cm^3^ g^−1^. These changes clearly indicated that the reaction environment during the introduction and grafting of hydroxamic acid groups onto the surfaces of channels reshaped the morphology of the channels, thereby causing a simultaneous decrease in the specific surface area, average pore size, and pore volume. This result was consistent with the morphological observations from the SEM images shown in Fig. 3. Although the functionalization process resulted in a decrease in the specific surface area and pore volume, A-PEA still retained mesoporous characteristics, ensuring unobstructed mass transfer channels. Furthermore, after adsorption, its structural parameters underwent pronounced changes. The BET specific surface area of the sample decreased to 9.59 m^2^ g^−1^, the average pore size dropped sharply to 6.06 nm, and the pore volume decreased to 0.009 cm^3^ g^−1^. These results implied that equilibrium adsorption was achieved despite the pore volume decreasing to 10% of that of pristine PEA. The adsorbed Ga(iii) not only occupied the channel space but likely also caused local blocking of the channels, particularly affecting the large-sized channels in the pore size distribution, thus leading to a reduction in the average pore size and a decline in the pore volume. The N_2_ adsorption/desorption isotherms and their corresponding pore size distribution curves are shown in Fig. 5, which reflected the aforementioned changing trends. It could be observed that the overall isotherm adsorption capacity decreased from PEA to A-PEA and dropped further after Ga(iii) adsorption. This reduction was more pronounced in the hysteresis loop at the relatively high-pressure region, indicating a transition of the porous structure from open to closed. The peak of the pore size distribution curve gradually shifted toward smaller pore sizes and became narrower, indicating a continuous reduction in pore size during the modification and adsorption processes. Consequently, the BET results revealed the evolution of the porous morphology of the PEA adsorbent and laid a foundation for subsequent studies investigating the adsorption mechanism, adsorption capacity, adsorption kinetics, and practical applications.

**Table 1:** BET porous structure parameters of PEA adsorbents

Sample	BET surface area (m^2^ g^−1^)	Pore size (nm)	Pore volume (cm^3^ g^−1^)
PEA	18.23	17.91	0.085
A-PEA	10.96	11.19	0.058
Adsorbed A-PEA	9.59	6.06	0.009

### 3.3 Adsorption kinetics in the simulated solution

**Fig. 6:**
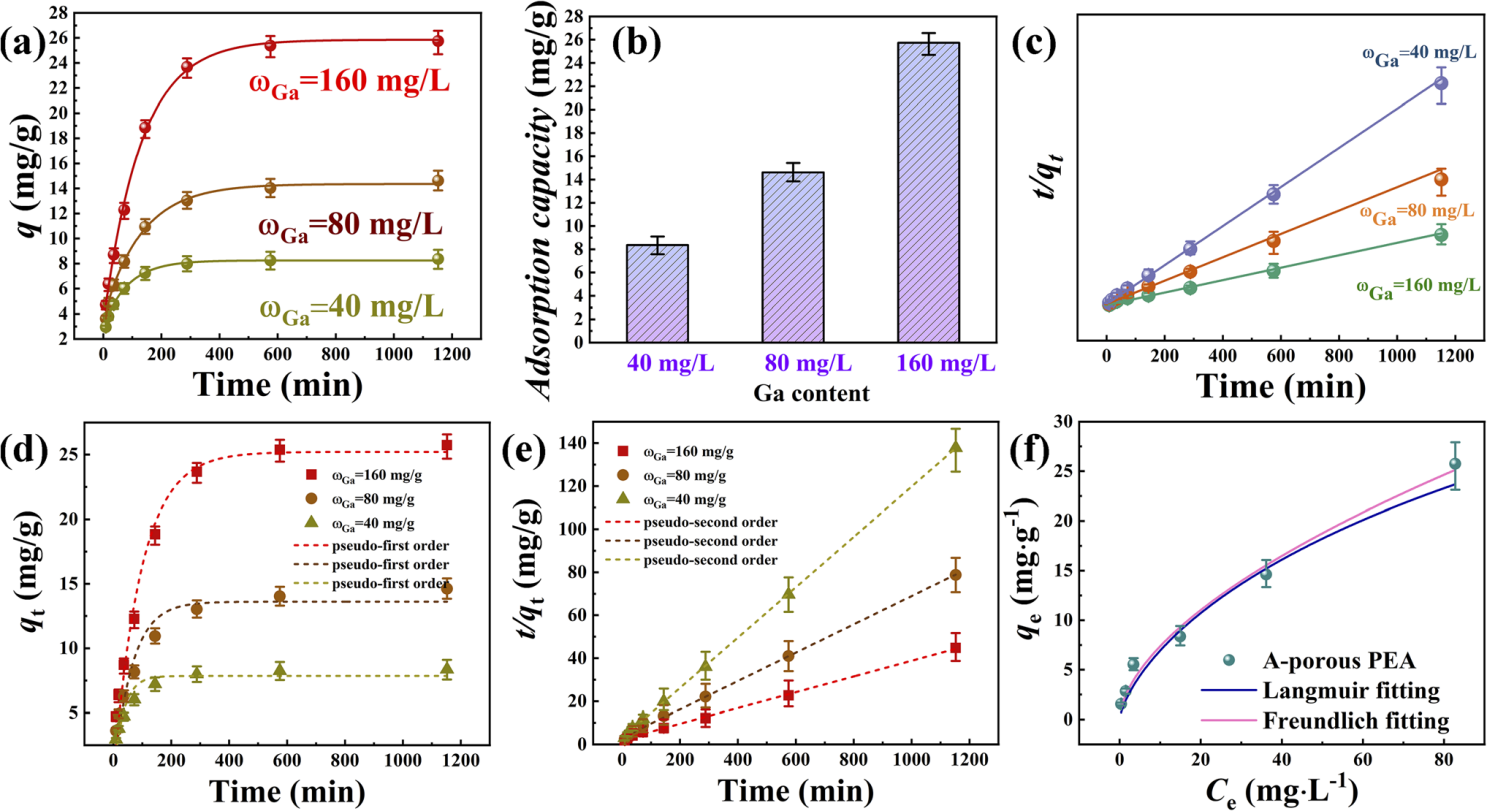
(a) Adsorption performance for Ga(iii), (b) equilibrium adsorption capacities for Ga(iii), (c) linear regression fitting of adsorption kinetics with different Ga(iii) concentrations, (d) pseudo-first-order and (e) pseudo-second-order linear regression fittings of adsorption kinetics with different Ga(iii) concentrations, and (f) equilibrium adsorption isotherms fitted with the Langmuir model and Freundlich model.

The pseudo-first-order kinetic model and pseudo-second-order kinetic model were employed to fit the adsorption processes in the simulated solutions with initial Ga concentrations of 160 mg L^−1^, 80 mg L^−1^, and 40 mg L^−1^. As shown in Fig. 6a–e and Table 2, the results demonstrated that the correlation coefficient (*R*^2^) of the pseudo-second-order kinetic model was higher than that of the pseudo-first-order kinetic model. It implied that the pseudo-second-order kinetic model could accurately describe the adsorption process of Ga. In the case of fitted equilibrium adsorption capacity (*q*_e_), the value fitted by the pseudo-second-order model was close to the actual adsorption capacity, whereas the values fitted by the pseudo-first-order model showed a large deviation from the actual situation. The pseudo-second-order kinetic model is based on the assumption that the adsorption rate is controlled by chemisorption, which involves electron transfer or chemical bonding between the active sites on the adsorbent surface and the adsorbate.^39^ Therefore, it could be inferred that the adsorption process of this porous adsorbent was mainly dominated by the chemisorption mechanism. Meanwhile, the adsorption rate constants *k*_1_ and *k*_2_ under different initial concentrations exhibited an obvious concentration dependence. As the initial Ga concentration increases from 40 mg L^−1^ to 160 mg L^−1^, the *k*_1_ value decreases from 0.02965 min^−1^ to 0.01054 min^−1^, and the *k*_2_ value decreases from 0.00491 min^−1^ to 0.00059 min^−1^. This phenomenon was attributed to the sufficient active sites in the adsorbent porous surface at low Ga concentrations. The adsorbate ions could quickly combine with the active sites, resulting in a larger adsorption rate constant. At high concentrations, the active sites were gradually occupied, and the competition among adsorbates was intensified, leading to a decrease in the adsorption rate and a decrease in the rate constant value.^40^ Combining the analysis results of the kinetic and isothermal adsorption models, it could be concluded that the adsorption process was mainly controlled by chemisorption. The porous structure of the A-PEA adsorbent provided abundant active adsorption sites due to the porous structure, and Ga(iii) was readily fixed on the porous surface of the A-PEA adsorbent.

**Table 2:** Fitting parameters of the pseudo-first-order and pseudo-second-order kinetic models of the porous A-PEA adsorbent

Concentration (mg L^−1^)	Pseudo-first-order kinetic model			Pseudo-second-order kinetic model		
	*q*_e_ (mg g^−1^)	*k*_1_ (min^−1^)	*R* ^2^	*q*_e_ (mg g^−1^)	*k*_2_ (min^−1^)	*R* ^2^
160	25.21	0.01054	0.9718	27.35	0.00059	0.9979
80	13.61	0.01562	0.9093	15.18	0.00140	0.9992
40	7.85	0.02965	0.9003	8.55	0.00491	0.9998

In addition, the Langmuir and Freundlich models were used to investigate the adsorption behavior of the A-PEA adsorbent. As shown in Fig. 6f and Table 3, the *R*^2^ of the Langmuir model was 0.9991, which was slightly higher than the *R*^2^ value of 0.9849 obtained from the Freundlich model. This trend implied that the Langmuir model was more suitable for describing the adsorption process. For the Langmuir model, it assumes that the adsorbent surface is homogeneous and the adsorption process is monolayer. The maximum adsorption capacity (*q*_m_) obtained by fitting the Langmuir model was 79.64 mg g^−1^, which demonstrated that this porous adsorbent had a high adsorption capacity for Ga and could effectively treat Ga-containing solutions with relatively high concentrations in practical applications.^41^ As shown in Table 3, the affinity constant (*K*_L_) was 0.01883 L mg^−1^, reflecting a moderate binding force between the adsorbent and Ga(iii). This affinity value not only ensured the effective capture of Ga(iii) by the adsorbent but also provided the possibility for subsequent desorption and recovery.^42^ Besides that, the *n* value of the Freundlich model was 1.723, which was higher than 1. This indicated that the adsorption process belonged to favorable adsorption, which implied that the adsorbent could efficiently adsorb Ga(iii) over a wide concentration range.^43^ It should be noted that the A-PEA adsorbent could still maintain excellent adsorption performance at low concentrations. However, the *K*_F_ value was 1.938 mg g^−1^. As an adsorption capacity constant, this value reflects the adsorption capacity of the A-PEA adsorbent for Ga(iii), where a larger *K*_F_ value is associated with a stronger adsorption capacity.^44^ In addition, when the initial concentration was 160 mg L^−1^, the equilibrium adsorption capacity reached 27.35 mg g^−1^, which was lower than the *q*_m_ value. This phenomenon indicated that the adsorbent approached adsorption saturation when treating Ga(iii) solutions at higher concentrations. In brief, the porous A-PEA adsorbent exhibited excellent chemisorption performance for Ga(iii) in simulated solutions and could be used in various real Bayer liquors. Given that Ga(iii) concentrations in real Bayer liquors varied across different regions, generally ranging from 10 to 300 mg L^−1^, the Ga(iii) concentration of the simulated solution used in this static adsorption study covered the main Ga(iii) concentration range in a typical real Bayer liquor.

**Table 3:** Fitting parameters of the Langmuir and Freundlich isotherm models for the porous PEA adsorbent

Metal	Langmuir model			Freundlich model		
	*q*_m_ (mg g^−1^)	*K*_L_ (L mg^−1^)	*R* ^2^	*n*	*K*_F_ (mg g^−1^)	*R* ^2^
Ga	79.64	0.01883	0.9991	1.723	1.938	0.9849

### 3.4 Adsorption kinetics in the real Bayer liquor

**Fig. 7:**
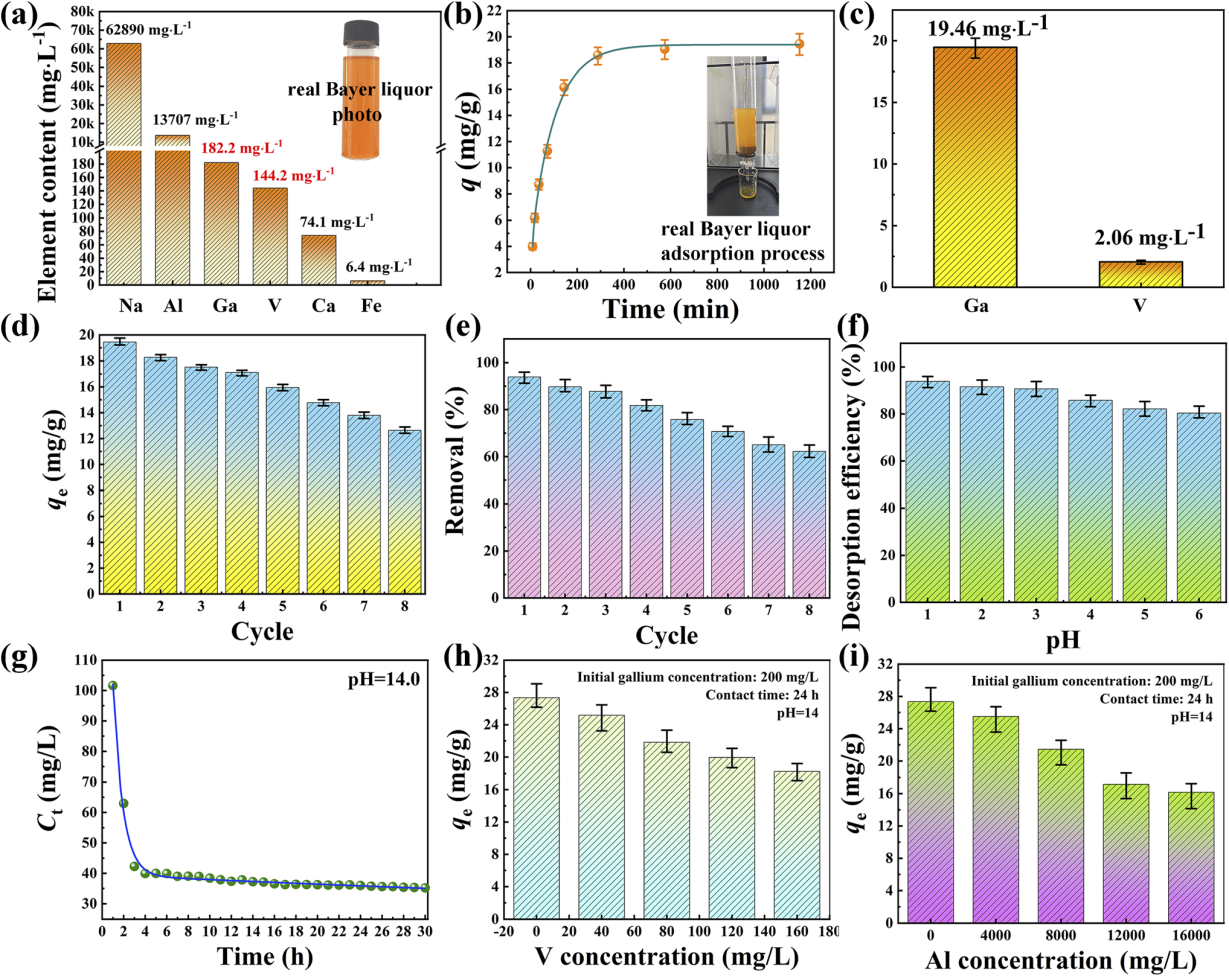
(a) Composition of the real Bayer liquor, (b) adsorption performance, (c) equilibrium adsorption capacities from the real Bayer liquor, (d) equilibrium adsorption values at different cycles, (e) removal values at different cycles, (f) desorption efficiency at different pH values, (g) real-time concentration of gallium in a solution at pH 14, and equilibrium adsorption values at different (h) V concentrations and (i) Al concentrations.

The performance of the porous A-PEA adsorbent in the real Bayer liquor is a critical factor for evaluating its practical application potential. Fig. 7a shows the contents of different ions in the Bayer liquor obtained from an aluminum plant (Zunyi city, Guizhou province, China) tested using ICP-OES. Sodium and aluminum ions are the dominant components in the real Bayer liquor, with concentrations of 62 890 mg L^−1^ and 13 707 mg L^−1^, respectively. In addition, besides the target Ga element with a concentration of 182.2 mg L^−1^, the liquor also contained a relatively high concentration of V ions, with a concentration of 144.2 mg L^−1^, and contained various elements such as Ca ions and Fe ions.

According to the previous adsorption data, the adsorption capacity of A-PEA for Ga in the simulated solution reached 25.21 mg g^−1^. It showed excellent adsorption potential under the simulated conditions. However, as shown in Fig. 7, after adsorption in the real Bayer liquor, the equilibrium adsorption capacity of Ga decreased to 19.46 mg g^−1^. The difference between the two systems was approximately 5.75 mg g^−1^, indicating a complex interaction between the simulated conditions and the real Bayer liquor environment. The real Bayer liquor had a pH value of 14. Hydroxamic acid groups might be fully deprotonated to form negatively charged hydroxamate anions, and Ga existed predominantly as the anionic gallate species [Ga(OH)_4_]^−^. The interaction was not electrostatic attraction between opposite charges. Instead, the adsorption proceeded through a ligand exchange mechanism. The deprotonated hydroxamate oxygen atoms might act as nucleophiles and replace the hydroxide ligands in [Ga(OH)_4_]^−^, forming stable five-membered chelate rings with the Ga(iii) center. This ligand substitution was thermodynamically favorable because hydroxamate ligands formed stronger coordinate bonds with Ga(iii) than hydroxide ions. The bidentate chelation through the O–O donor set of the hydroxamate group overrides the electrostatic repulsion between the anionic species.

In such complex metal cation coexisting systems, the adsorption capacity of Ga was still high. According to the adsorption kinetic curve shown in Fig. 7b, the adsorption capacity of Ga increased with the increase in contact time, rapidly rising to 18.59 mg g^−1^ within 288 min, and then reached an equilibrium state. This trend conformed to the typical kinetic characteristics of mass transfer and surface reaction in the adsorption process. Considering that the hydroxamic acid groups have a strong binding ability for high-valent metal cations such as Ga(iii) and V(v), the presence of V(v) was highly likely to directly compete with Ga(iii) for the adsorption active sites. As shown in Fig. 7c, the equilibrium adsorption capacity of V(v) was 2.06 mg g^−1^, which proved that V was adsorbed by A-PEA simultaneously, occupying 12.5% of the functionalized sites, thus directly explaining the decrease in the Ga adsorption capacity. In another aspect, the real Bayer liquor has a complex cation composition, and its high Na ion concentration might change the morphological distribution of Ga(iii) through the salt effect or the electrostatic shielding effect, further affecting the adsorption efficiency. Despite the competitive adsorption, porous A-PEA could still achieve a Ga adsorption capacity of 19.46 mg g^−1^ in the real Bayer liquor, which indicated that its structural design strategy was effective for practical applications. To assess the recycling performance of the absorbent, A-PEA saturated with adsorbed Ga(iii) was first rinsed with water and then immersed in a 0.1 mol per L HCl solution for 1 hour to facilitate desorption. Subsequently, the absorbent was washed with water again, and the adsorption–desorption cycle experiment was repeated. The results are presented in Fig. 7d. It could be observed that the saturated adsorption capacity of Ga(iii) declined as the number of cycles increased. In the initial stage, the saturation adsorption capacity of A-PEA did not exhibit a significant decrease. At the eighth cycle, the saturation adsorption capacity of A-PEA for Ga(iii) was 64.98% of the initial adsorption capacity. This was attributed to a combination of the incomplete elution of strongly bound Ga(iii) species during each desorption step and partial degradation or protonation-induced conformational changes of the hydroxamic acid functional groups under repeated exposure to strong acid. Nevertheless, the adsorption capacity of A-PEA in the third cycle was as high as 90.13% of its initial value, indicating its robust stability and potential for reuse.

Fig. 7e and f depicts the removal efficiency of Ga(iii) by A-PEA at different cycle numbers and the desorption efficiency of Ga(iii) at different pH values, respectively. As the pH of the solution increased from 1 to 6, the desorption efficiency of Ga(iii) decreased gradually from 93.88% to 80.36%. This phenomenon indicated that the optimal desorption efficiency occurred with a 0.1 mol L^−1^ HCl eluent. In the actual Ga(iii) extraction process, the A-PEA adsorbent was used under strongly basic conditions. Hence, alkali resistance is a crucial property for the A-PEA adsorbent. The A-PEA adsorbent was investigated in a strong-alkali solution before and after soaking in the adsorption solution for 24 hours, as presented in Fig. 7g. Fig. 7h and i shows the equilibrium adsorption capacities of A-PEA for Ga(iii) with different V and Al concentrations, which decreased with an increase in the concentration of V or Al. This could be attributed to the coordination of V with the hydroxamic acid groups, which occupied the adsorption sites.^45^ When the V and Al concentrations reached 160 mg L^−1^ and 16 000 mg L^−1^, respectively, the corresponding loss in the equilibrium adsorption capacities of A-PEA for Ga(iii) was 36.56% and 40.87%, suggesting that the material still retained a certain degree of adsorption selectivity for Ga(iii) despite the highly competitive matrix.

## 4 Conclusions

In this study, a high-efficiency adsorbent with an oriented porous structure was prepared by combining the ice-templating method with hydroxamic acid modification. The adsorbent had a specific surface area of 10.96 m^2^ g^−1^, and the oriented porous adsorbent was functionalized *via* a nucleophilic addition reaction. The N content on the inner surface of the channels reached 14.65 at%, as determined by XPS analysis. The highly overlapping distribution of Ga and N elements determined using EDS mapping further verified the specific coordination adsorption mechanism between hydroxamic acid groups and Ga(iii). Meanwhile, its equilibrium adsorption capacity for Ga in the simulated solutions reached 27.35 mg g^−1^, with a maximum theoretical adsorption capacity of 79.64 mg g^−1^. Adsorption kinetic analysis indicated that the adsorption process was mainly dominated by a chemisorption mechanism, and the fitting results of the adsorption isotherm implied that the process exhibited homogeneous monolayer adsorption characteristics. However, when the adsorbent was applied to the real Bayer liquor containing a complex matrix of coexisting cations, the equilibrium adsorption capacity for Ga decreased to 19.46 mg g^−1^ due to the competitive adsorption of V and the influence of a high-salinity environment. Despite the reduction in capacity, the adsorbent retained approximately 87.5% of its original adsorption capacity in the complex industrial liquor. Overall, this work provides a feasible route for the industrial recovery of gallium from Bayer liquor and can be extended to the design of other high-performance adsorbents for rare metal separation.

## Author contributions

L. Ren conceived the research idea and drafted the manuscript. W. Liu and X. Wu carried out the synthesis experiments and data analysis. Y. Tu performed the adsorption tests. Z. Yao analyzed the adsorption properties. Y. Li supervised the whole work and revised the manuscript. All authors read and approved the final manuscript.

## Conflicts of interest

There are no conflicts to declare.

## Supplementary Material

RA-OLF-D6RA05324F-s001

## Data Availability

This article contains no supplementary data. All data relevant to this study are included within the manuscript. Supplementary information (SI): the comparison of the adsorption performance for different adsorbents, comparison of the adsorption performance for porous adsorbent and dense solid adsorbent, and the XPS analysis of the A-PEA adsorbent. See DOI: https://doi.org/10.1039/d6ra05324f.
