# Supplementary material for: Gallium extraction from Bayer liquor using hydroxamic acid-functionalized poly(ethyl acrylate) adsorbents with oriented porous structures
Source: RSC Adv. 2026 Aug 19;16(41):48869–81. doi: 10.1039/d6ra05324f (PMC13487455; doi:10.1039/d6ra05324f)
Supplement: Supplementary file 1 [file RA-016-D6RA05324F-s001.pdf]

Supplementary Information (SI) for RSC Advances.
This journal is © The Royal Society of Chemistry 2026

Supplementary Information for

Gallium Extraction from Bayer Liquor Using Hydroxamic Acid-Functionalized Poly(ethyl acrylate) Adsorbents with Oriented Porous Structures

*Li Ren, Wei Liu, Xian Wu, Yanbin Tu, Zhengjie Yao, Yang Li**

School of Materials and Energy Engineering, Guizhou Institute of Technology, Guiyang, China

**Corresponding author. E-mail: 20130214@git.edu.cn*

Comparison of the adsorption performance for different adsorbents

Table S1 showed the comparison of the adsorption performance for different adsorbents. It systematically compared the saturated adsorption capacity, pH, raw material cost and references of diverse Ga adsorbents. Commercial D201 and IRA900 resins fit pH 14 alkaline Bayer liquor but showed extremely low Ga adsorption, failing industrial demands. LSC600 worked at pH 13.7 with a capacity of 3.6 $mg \cdot g^{-1}$ and costed 14–16 RMB/kg, yet its adsorption capacity remained unsatisfactory. Mesoporous carbon and amide-cellulose only function in acidic solutions, requiring extra acid neutralization that increases process costs. MOFs achieved 35.4 $mg \cdot g^{-1}$ and surpass traditional adsorbents, but their cost of 1000–3000 RMB/kg made mass production uneconomical. COFs possessed the highest capacity of 687.38 $mg \cdot g^{-1}$ at pH 6.5 with a prohibitive cost of 1000–5000 RMB/kg, unsuitable for strongly alkaline Bayer liquor. Our adsorbent stably operated at pH 14 with a capacity of 19.46 $mg \cdot g^{-1}$, much higher than alkali-resistant commercial resins and acid-type carbon/cellulose materials, only slightly lower than MOFs. Its raw material cost of 5–10 RMB/kg was far cheaper than MOFs and COFs. Integrating excellent alkali tolerance, favorable adsorption capacity and low cost, the maximum adsorption capacity of this work was over 102 times larger than that of D201. This material met industrial Ga extraction standards and overcame the universal defect of existing adsorbents, poor capacity for alkali-stable materials or high costs and weak alkali resistance for high-capacity porous adsorbents.

Table S1 Comparison of the adsorption performance for different adsorbents

**Adsorbent**	q_e **($mg \cdot g^{-1}$)**	**pH**	**Cost (RMB/kg)**	**Ref.**
D201	0.19	14.0	5-10	[1]
IRA900	0.11	14.0	18-20	[1]
LSC600	3.6	13.7	22-30	[1]
mesoporous carbon	4.4	3.0	12-25	[2]
amide-cellulose	3.4	3.0	15-20	[3]
MOF	35.4	3.0	1000-3000	[4]
COF	687.38	6.5	1000-5000	[5]
this work	19.46	14.0	5-10	-

1 Z. Qin, S. Wang, S. Zhang, J. Xie, C. Zhou, C. Wang, L. Song, K. Ma, D. Luo, H. Yue. Cross-linked amidoxime porous resin for selective gallium separation in Bayer solutions: reaction mechanism and kinetic study. Chemical Engineering Journal, 2024, 481: 148340.

2 E. Díez, J.M. Gomez, A. Rodríguez, I. Bernabe, J. Galan. Recovery of Gallium from Aqueous Solution through Preconcentration by Adsorption/Desorption on Disordered Mesoporous Carbon. Journal of Sustainable Metallurgy, 2021, 7: 227.

3 W. Guo, J. Zhang, F. Yang, F. Tan, Z. Zhao. Highly efficient and selective recovery of gallium achieved on an amide-functionalized cellulose. Separation and Purification Technology, 2020, 237: 116355.
4 X. Cai, Y. Li, H. Wang. Defective UiO-66-NH_2 for simultaneous adsorption of phosphate and Pb(II): mechanistic insights into Zr–O–Pb interactions. Inorganic Chemistry, 2024, 63: 7021.
5 B. Zhang, H. Zheng, K. Yang, C. Li, T. Wu, Q. Sui, W. Feng. Bottom-up synthesis of a sulfhydryl-modified heteroporous covalent organic framework for ultrafast removal of trace Hg(II) from water. Chemosphere, 2024,360: 142410.

Comparison of the adsorption performance for porous adsorbent and dense solid adsorbent

The non-frozen PEA sample dried under ambient conditions was prepared, resulting in a non-porous, non-oriented structure. Under a uniform initial Ga concentration of 160 mg/L, the effect of adsorbent structure and morphology on Ga adsorption performance was investigated as shown in **Figure S1**. The adsorption experimental results revealed a significant difference in Ga adsorption capacity between the two adsorbents. The freeze-dried porous adsorbent exhibited an adsorption capacity of 25.74 mg/g, while the dense solid adsorbent only achieved 3.16 mg/g. The test results confirmed that the porous structure could remarkably improve the Ga adsorption performance of adsorbents, which was consistent with general theoretical understanding. The porous structure constructed via freeze-drying effectively increased the specific surface area, enriched the pore structure, and exposed abundant surface-active sites, providing sufficient attachment sites and diffusion channels for gallium ions. In contrast, the dense solid adsorbent featured a compact structure and low specific surface area, which limited the number of available active sites and reduces ion diffusion efficiency, thereby severely restricting its adsorption performance.

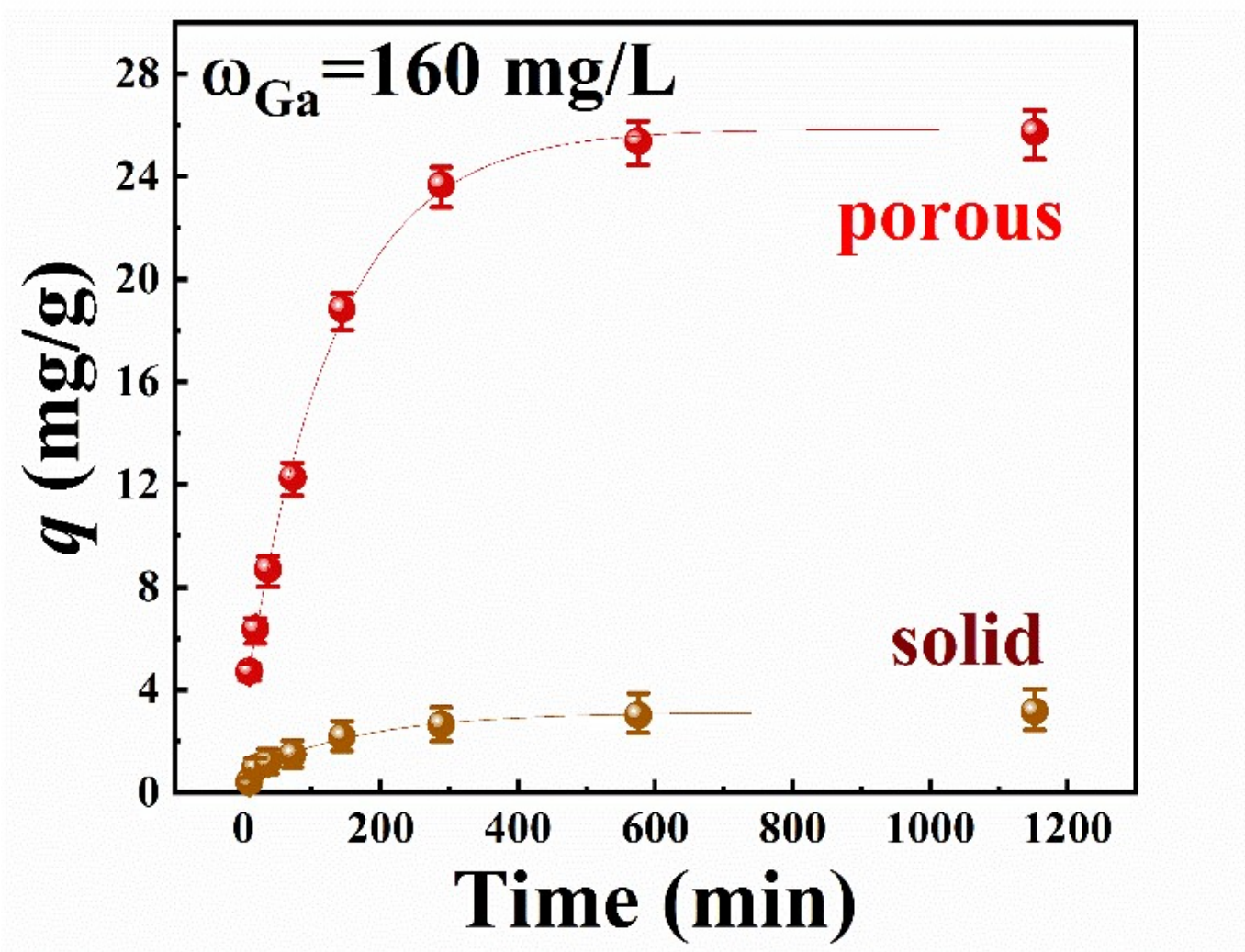

Figure S1 The adsorption performances for porous adsorbent and dense solid adsorbent

The XPS analysis of the A-PEA adsorbent

Figure S2 displayed the XPS spectra of adsorbent A-PEA. The XPS survey spectrum clearly identified characteristic photoelectron signals of C, O and N elements, which directly verified the successful introduction of nitrogen-containing functional groups onto the material surface. The high-resolution C1s spectrum exhibited a dominant peak at 284.83 eV assigned to C–C bonds in the carbon skeleton, which served as the reference for charge calibration. Carbon accounts for 74.05 % and constituted the primary component of the substrate. The O1s high-resolution spectrum showed a characteristic peak at 531.26 eV corresponding to ester groups on the material surface, with an atomic percentage of 11.30%. A sharp, high signal-to-noise ratio N1s peak emerged at 399.17 eV, which was attributed to adsorption active sites. Quantitative analysis revealed that nitrogen reached an atomic percentage of 14.65%, strongly demonstrating abundant grafting of nitrogen-modified groups on the adsorbent surface and confirming the successful amino-functionalization modification. The rich nitrogen-containing active sites could provide sufficient chelation adsorption sites for target pollutants, laying a crucial structural foundation for outstanding adsorption capacity. The atomic ratios of C, N and O further indicated that the carbon substrate framework remains intact during modification, while nitrogen-containing adsorption groups were efficiently immobilized.

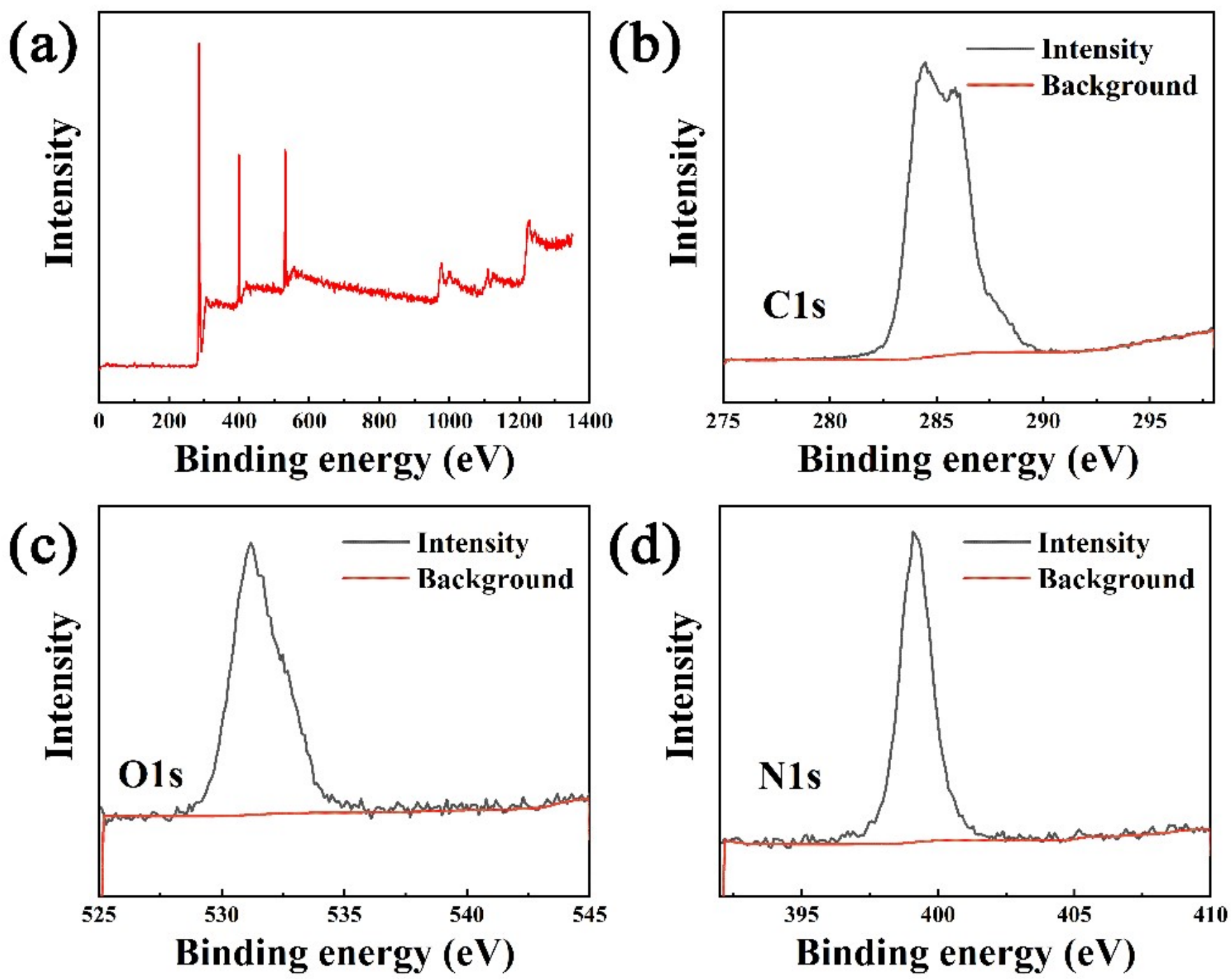

Figure S2 High-resolution (a) XPS survey, (b) C1s, (c) O1s, and (d) N1s spectra of A-PEA

Table S2 The peak table of XPS spectra

Name	Start	Peak	End	Height	FWHM	Area (P)	Area (N)	Atomic
	BE	BE	BE	CPS	eV	CPS.eV	TPP-2M	%

C1s	297.98	284.83	275.18	18175.63	3.12	62678.52	878.92	74.05
N1s	409.98	399.17	392.18	11607.95	1.32	19253.55	173.90	14.65
O1s	544.98	531.26	525.18	8423.49	2.48	23130.83	134.12	11.30
